# Allogeneic CART progress: platforms, current progress and limitations

**DOI:** 10.3389/fimmu.2025.1557157

**Published:** 2025-06-12

**Authors:** Ameneh Shokati, Maryam Sanjari-Pour, Mahshid Akhavan Rahnama, Saghar Hoseinzadeh, Mohammad Vaezi, Mohammad Ahmadvand

**Affiliations:** ^1^ Department of Applied Cell Sciences, School of Advanced Technologies in Medicine, Tehran University of Medical Sciences, Tehran, Iran; ^2^ Department of Clinical Biochemistry, Faculty of Medical Science, Tarbiat Modarres University, Tehran, Iran; ^3^ Cell Therapy and Hematopoietic Stem Cell Transplantation Research Center, Research Institute for Oncology, Hematology and Cell Therapy, Tehran University of Medical Sciences, Tehran, Iran; ^4^ Department of Medical Biotechnology, Faculty of Advanced Medical Sciences, Tabriz University of Medical Sciences, Tabriz, Iran

**Keywords:** allogeneic CAR T cells, editing technology, non-editing technology, off-the-shelf CART cell, graft-versus-host disease

## Abstract

Allogenic chimeric antigen receptor T (CAR-T) cells have advantages compared to autologous T cell therapies such as availability cells for production, a suitable HLA-matched donor (if graft-vs-host-disease and rejection effects are to be avoided and also lower risks associated with transduction methods in process of autologous CAR-T cells). In recent years, the additional editing and non-editing technologies are helping to make allogenic CAR-T therapies a hopeful future treatment. Universal off-the-shelf CAR-T cells can be solved key issues include preventing graft-versus-host disease (GVHD) and time consumption and other challenges faced to allogenic CAR-T cells. Here, we have highlighted the improvement in CAR-T development, particularly in engineering allogenic CAR-T, clinical practices related to these, pre-clinical and clinical studies and their successes which investigated in recent 10 years related to treatment of hematological malignancies and cancers by allogenic CAR-T cells.

## 1 Introduction

**Figure 1:**
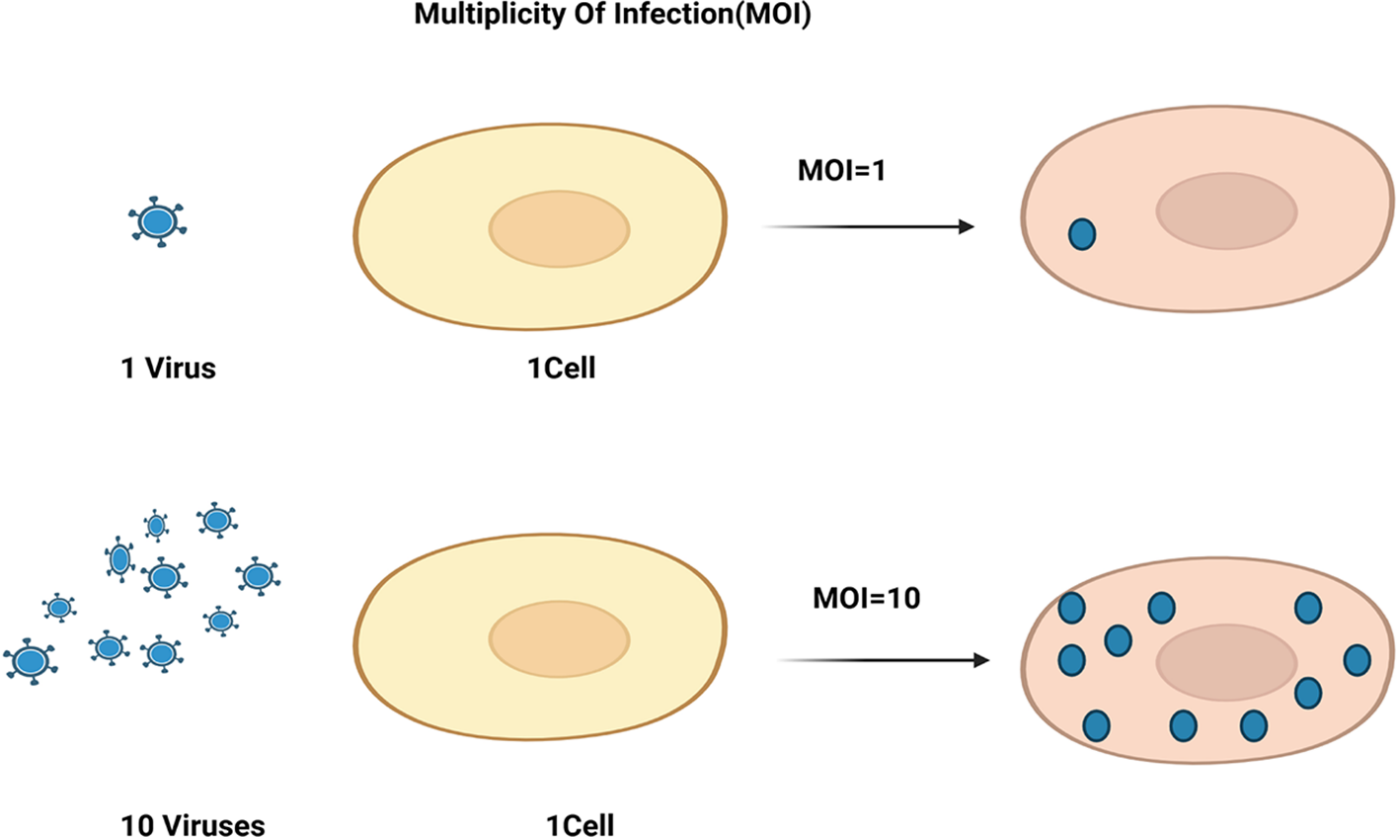
Multiplicity of infection (MOI) represents the ratio of infectious agents, typically viral particles, to target cells in a defined system, such as a cell culture. For instance, adding 10 million viruses to 1 million cells results in a MOI of 10, indicating an average of 10 viral particles per cell.

Recent advancements in molecular and cellular biology, coupled with an enhanced understanding of the function and nature of tumors, have led to several innovative strategies in targeted immunotherapy. Key therapeutic approaches in this domain now encompass monoclonal and bi-specific antibodies, antibody-drug conjugates, checkpoint inhibitors, and the latest advancements in adoptive cell therapy (ACT). Over the past decade, CAR-T immunotherapy has emerged as a pioneering approach to ACT. CAR-T cell therapy is not only used for cancer treatment but is also being investigated for various diseases, broadening the potential applications and impacts in the medical field. This innovative form of immunotherapy has demonstrated exceptional efficacy in treating hematopoietic cancers and shows considerable promise for addressing the challenges associated with solid tumors (1, 2). A CAR is a synthetic construct that comprises an extracellular, a spacer or hinge region, a transmembrane domain, and one or more intracellular signaling domains, expressed on the T cell surface and other immune cells through non-viral or viral transduction recognizing and interacting with tumor-associated antigens (3). In addition, to overcome the limitation of lentiviral transduction in primary T cells, scientists use high multiplicities of infection (MOI) of the virus by standard protocols. As shown in **Figure 1**, MOI represents the ratio of infectious agents, typically viral particles, to target cells in a defined system, such as a cell culture. The use of high MOI can affect the success of process through increasing both the time and cost of producing CAR-T cells (4).

**Figure 2:**
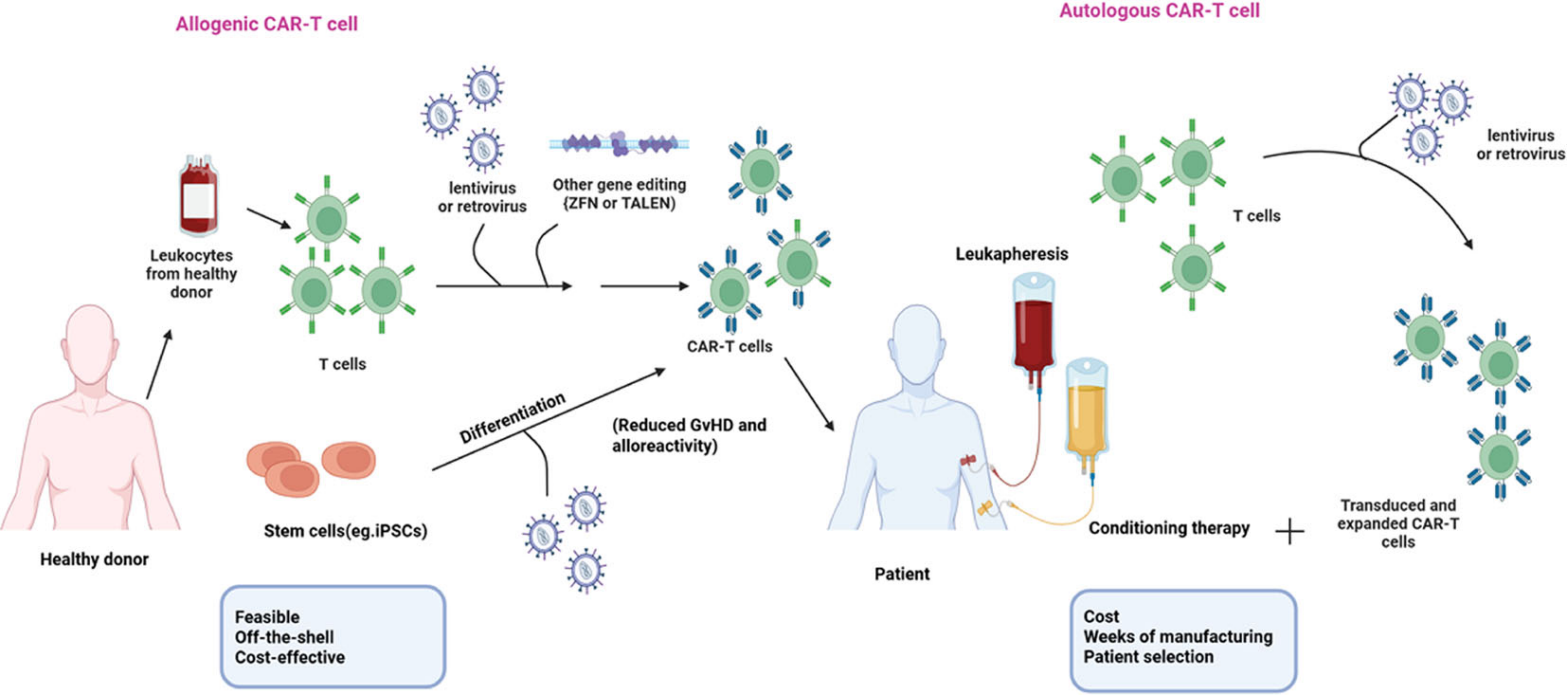
Autologous vs. Allogeneic CAR-T cell therapy comparison.

According to different purposes, the design of CAR-T cells varies significantly, requiring careful consideration and optimization of multiple factors to achieve optimal therapeutic outcomes. Once the CAR genes are successfully incorporated into the T-cell genome, the modified T cells are expanded to achieve the desired therapeutic dosage. The expanded CAR-T cells are administered to the patient, leverage the patient’s own, and employ genetically engineered T cells to direct potent therapeutic responses to target and eradicate cancer cells specifically (5). To date, most FDA-approved CAR-T cell products are derived from autologous immune cells including Tisagenlecleucel (Kymriah, Novartis) highly effective in treating patients with relapsed or refractory CD19-positive hematological malignancies, Axicabtagene ciloleucel (Yescarta, Kite Pharmaceuticals)Approved for the treatment of large B-cell lymphoma, Brexucabtagene autoleucel (Tecartus, Kite Pharmaceuticals) Targets B-cell acute lymphoblastic leukemia (ALL) and mantle cell lymphoma (MCL), Idecabtagene vicleucel (Abecma) and Carvykti^®^ (ciltacabtagene autoleucel) used for the treatment of relapsed or refractory multiple myeloma, Breyanzi^®^ (lisocabtagene maraleucel) for second-line treatment of large B-cell lymphoma (6). These therapies have demonstrated significant efficacy in their respective indications, providing new treatment options for patients with limited therapeutic choices. Although the success of currently approved autologous CAR-T cell products can cure up to 35-40% of patients (7) and have a lower risk of immunologic incompatibility between donor and recipient, their widespread application faces significant clinical and economic challenges. The complex manufacturing process for these therapies begins with collecting peripheral blood mononuclear cells (PBMCs) from each patient through leukapheresis. T cells are genetically engineered to produce CAR-T cells with enhanced antitumor functions, subsequently reinfused into the patient. Treatment preparation is a time-consuming process that typically takes about three weeks, which can be particularly problematic for patients with rapidly advancing conditions, such as acute leukemia. Additionally, patients often undergo lymphodepletion through chemotherapy and radiotherapy, making it challenging to collect T cells that meet the required standards for both quantity and quality and can result in a manufacturing failure rate of 2% to 10%. Also, the effectiveness of CAR-T cells can be diminished by T cell exhaustion, a common T cell dysfunction observed in cancer patients (8–10). On the other hand, the heterogeneity in tumor antigen expression and the immune evasion strategies employed by tumor cells, necessitate the use of CAR-T cell products that can target multiple antigen specificities. Finally, autologous cell therapy is designed for individual patients, incurring significant costs and restricting its broader application allogenic CAR T-cell therapy, sourced from healthy donors who are human leukocyte antigen (HLA)-matched or gene-edited cells modified for use in non-HLA-matched patients, offers several advantages over autologous approaches (11). Healthy donors provide a high yield of cells from a single individual, and their PBMCs are in optimal condition, having not been subjected to chemotherapy or radiotherapy. The immediate availability of cryopreserved batches and the potential for standardizing CAR-T cell products allow multiple modifications and combinations to target various tumor antigens, leading to more predictable outcomes. This “off-the-shelf” immunotherapeutic approach enables a single manufacturing run to produce doses for multiple patients or multiple doses for a single patient. By scaling up production and establishing a bank of CAR immune cells from healthy donors, the cost per patient can be reduced, and access to the therapy can be broadened, simplifying supply chain logistics and eliminating the need for bridging therapies (12, 13). **Figure 2** compares two steps in autologous and allogeneic CAR-T cell therapy, including cell sourcing and genetic modification.

Autologous CAR-T therapy uses a patient’s T cells, genetically modified to express a CAR, minimizing immune rejection but facing challenges like high cost, time delays, and variable T cell quality. Allogeneic CAR-T therapy, sourced from healthy donors or iPSCs, offers scalable, off-the-shelf treatment with faster delivery but risks complications like GvHD and immune rejection. Genetic modifications, including TCR disruption, HLA ablation, and NK cell inhibitory ligand overexpression, aim to reduce these risks and improve persistence and efficacy.

Allogenic CAR-T cell therapy faces significant challenges. One major issue arises when the T-cell receptor (TCR) on the surface of allogenic CAR-T cells recognizes and attacks the patient’s healthy tissues, leading to GvHD risk. Additionally, host-versus-graft (HvG) reactions can occur, in which the patient’s immune system identifies the CAR-T cells as foreign and eliminates them. To address these issues, researchers can choose T-cell sources with lower TCR signaling capacity to minimize them. Furthermore, CAR-T cells genetically edited (using ZFN, TALEN, CRISPR/Cas9) are being developed to reduce the risk of immune rejection and improve their effectiveness. However, these technologies come with significant challenges and limitations, such as Double-Strand Breaks (DSBs) in DNA leading to unintended mutations, genomic instability, potential safety risks, and lack of modulation resulting in off-target effects and unpredictable outcomes (14–16). Also, CAR T-cell therapy can have several side effects. Immediate effects include cytokine release syndrome (CRS) and immune effector cell-associated neurotoxicity syndrome (ICANS) and long-term side effects can involve hypogammaglobulinemia and cytopenias (17). Despite these obstacles, continuous research and progress in gene-editing technologies show promise for enhancing the safety and efficacy of allogenic CAR T-cell therapies. In this review, we aim to provide a thorough overview of the latest advancements and challenges in CAR-T cell therapy, its clinical applications, and recent innovations in gene editing technologies. We will also discuss the challenges and side effects associated with CAR-T cell therapy, along with strategies to mitigate these effects. Our focus is to highlight the remarkable potential of CAR-T cell therapy to transform cancer treatment and significantly improve patient outcomes.

## 2 Source of allogeneic cells

Given the rising demand for CAR therapy, exploring novel immunotherapy strategies is crucial. Both autologous and allogeneic T cells face limitations, including restricted expansion and exhaustion. Allogeneic CAR-T cells also pose a risk of GvHD. While PBMCs are the primary source for allo-CAR-Ts, iPSCs, and umbilical cord blood offer a scalable, antigen-specific T-cell supply with optimized therapeutic features, addressing these challenges.

### 2.1 Peripheral blood mononuclear cells

PBMCs are typically obtained from the blood of healthy donors, presenting an opportunity to create a cell bank that includes various subtypes of the HLA complex. It allows for selecting batches that match the HLA types to produce multiple vials from a single apheresis product, facilitating a rapid and standardized manufacturing protocol (18).

### 2.2 Umbilical cord blood cells

While there are challenges, such as the limited availability of UCB cells, their benefits are substantial. UCB cells are a valuable source of hematopoietic stem cells (HSCs) with distinct advantages over adult T cells. UCB cells are particularly “antigen-naïve,” which reduces their alloreactivity and allows for greater flexibility in HLA matching (19). Additionally, reduced NFAT signaling and lower NF-κB activation lead to decreased production of pro-inflammatory cytokines, which helps reduce the frequency and severity of GvHD (20). Furthermore, UCB cells exhibit much lower exhaustion markers like PD-1, TIM-3, and LAG-3 than peripheral blood T cells, enhancing their long-term persistence and effectiveness (21).

### 2.3 Induced pluripotent stem cells

iPSCs can proliferate indefinitely while retaining pluripotency, enabling the generation of diverse, genetically modified CAR-T cells with superior proliferation capacity and longer telomeres than mature T cells. This feature allows for the feasible generation of a diverse range of genetically modified CAR-T cells, which enhances antitumor effectiveness, significantly reduces immunogenicity, improves compatibility, and effectively mitigates the risk of allorejection (22).

**Figure 3:**
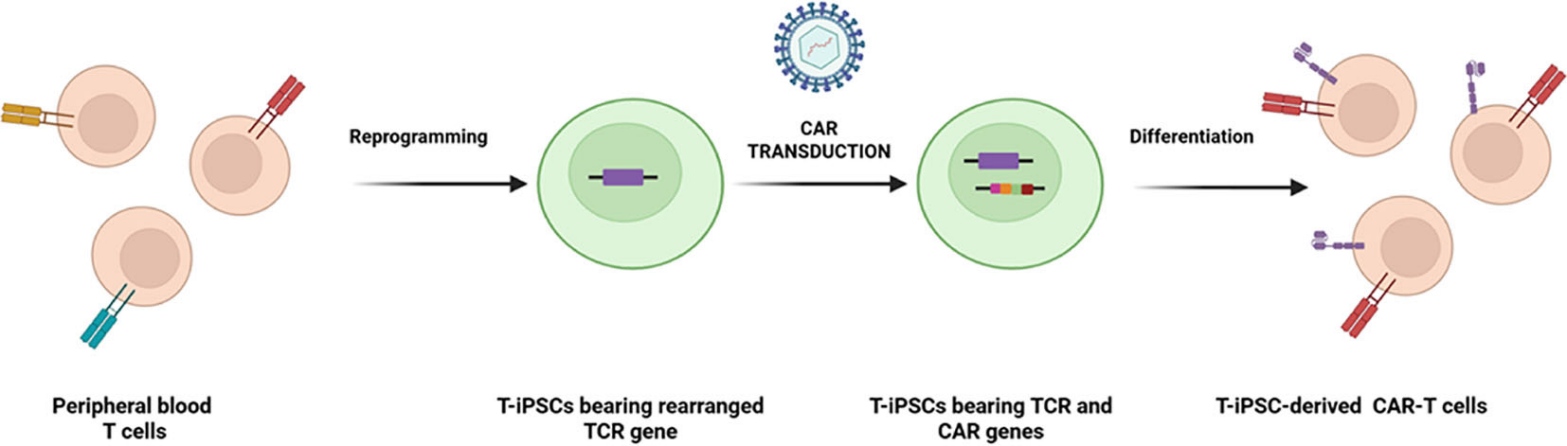
Generation of iPSC-derived T cells from peripheral blood T Cells.

The generation of iPSC-derived CAR-T cells from adult somatic cells generally involves two key steps:(a) Reprogramming somatic cells into iPSCs via the introduction of transcription factors such as Oct4, Sox2, Nanog, Lin28, Klf4, and c-Myc (23), and TCR modification to generate hypoimmunogenic iPSC lines;(b) Genetic engineering, where CAR transgenes are introduced into iPSC-derived T cells to direct tumor-specific targeting (**Figure 3**) (24).

A significant advantage of iPSCs is that CAR-T cells can be derived from a single clone, allowing for uniform clonal expansion and stable genetic modifications for reliable and effective treatment. In this regard, Jing et al. successfully generated mature T cells from human iPSCs using G9a/GLP inhibition and exhibited characteristics similar to mature alpha-beta T cells from peripheral blood, including effector and memory-like subpopulations (25). Kinoshita et al. introduced GD2-CARrejTs, a new iPSC-derived CAR-T cell therapy for small cell lung cancer (SCLC). These rejuvenated T cells showed more potent cytotoxic effects than conventional GD2-CARTs (26). In the realm of clinical applications, fate therapeutics is a leading company developing off-the-shelf iCAR-T products for various therapies, and its products, including FT819 (anti-CD19) and FT825/ONO-8250 (anti-HER2), are currently in Phase I clinical trials (27, 28). While stem cell-based approaches hold great promise, their successful clinical application will depend on ongoing innovation and rigorous evaluation.

Peripheral blood T cells, retaining their rearranged TCR gene, can be reprogrammed into iPSCs. These T-iPSCs can be engineered with CAR for enhanced tumor specificity in adoptive cell therapy or derived from antigen-specific T cell clones to produce targeted T cells.

## 3 Mechanisms of action: how allogenic CAR-T cells work

When human T cells are equipped with a CAR, they can effectively eradicate tumor cells in a MHC- and Fas-independent manner, making them versatile and powerful in targeting cancer cells (29). CAR-T cells engage with target cells by creating a unique immunological synapse. Once the immunological synapse is established, the CAR-T cells can induce tumor cell lysis through various pathways (30).

**Figure 4:**
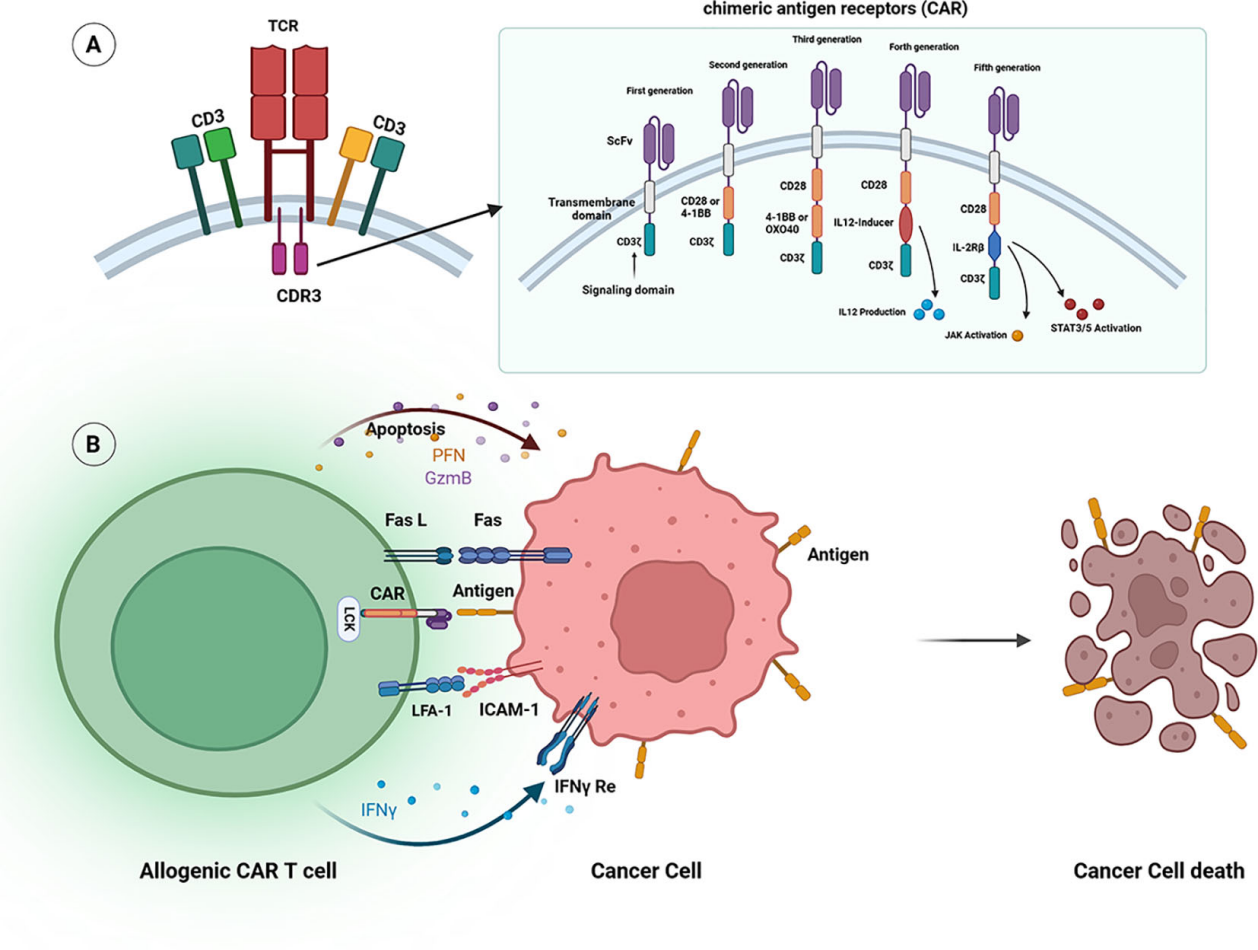
**(A)** Structure of CAR T-cells. The structure of Chimeric Antigen Receptors (CARs) in CAR T-cells comprises several essential components: a single chain variable fragment (scFv), a hinge region along with a transmembrane (TM) region, costimulatory domains (such as 4-1BB, ICOS, CD28, and OX40), and a signaling domain (CD3ζ). First-generation CARs included only CD3ζ as the intracellular domain. In contrast, second- and third-generation CARs feature one or two costimulatory domains connected to CD3ζ, respectively. Fourth-generation CARs, referred to as “TRUCK” CARs, are structurally similar to second-generation CARs but incorporate an inducible cytokine expression (such as IL-12) via an NFAT-responsive promoter. Fifth-generation CAR-T cells include a JAK/STAT activation domain derived from IL-2RB, positioned between CD28 and CD247. This addition promotes cells proliferation, prevents terminal differentiation, and enhances persistence. **(B)** The mechanism of action of CAR T-cells involves the binding of CARs to specific antigens present on the surface of tumor cells through the scFv recognition domain. This interaction triggers anticancer effects by promoting the inflammatory cytokines’ secretion, such as IFN-γ, and facilitates cytolytic effector function via granzyme and perforin in an ICAM-1-dependent manner.

CAR-T cells primarily achieve tumor lysis through direct interactions with tumor cells. CAR-T cell initial activation was strongly dependent on the expression level of ICAM1 Cytokine production by activated CAR-T cells,enhances their anti-tumoral capabilities and is crucial in mediating tumor lysis via secondary mechanisms (31). CAR-T cells exhibit a disorganized lymphocyte-specific protein tyrosine kinase (Lck) arrangement and recruit lytic granules more quickly than TCRs, leading to a more rapid killing of tumor target cells and faster detachment from dying tumor cells by CAR-T cells (32). The perforin pathway and FasL (Fas Ligand) pathway can collaborate to kill target cells. This synergy is essential for achieving complete and durable tumor control by CAR-T cells (shown in **Figure 4**) (33).

CAR-T cell therapies exhibit greater sensitivity to target antigens than antibody or antibody-drug conjugate therapies. They can detect and respond to lower levels of antigens present in tumor cells (34). In dealing with a tumor that changes over time, multifunctional CAR-T cells, which can be bispecific, switchable, or capable of delivering other therapeutic agents directly, can perform multiple actions simultaneously and be effective (35, 36).

## 4 Food and drug administration approval of CARTs

Since 2017, the FDA has authorized six CAR-T cells in the USA and other nations, and the National Medical Products Administration of China has approved two CAR-T products. These products target patients suffering from acute lymphoblastic leukemia (ALL), multiple myeloma (MM), or advanced/resistant large B-cell lymphoma (LBCL), in which significant results are acquired with total response rates that may reach as high as 100% measurable response rates in a number of cases (37, 38).

It was for the first time that the FDA approved CAR-T cells therapy on Aug 30th, 2017, to treat acute lymphoblastic leukemia in young adults and children. Later, the FDA approved three additional CD19-specific CAR-T cells for the purpose of treating various B cell malignancies, i.e., Breyanzi, Tecartus, and Yescarta. Even though the CAR expressed in Tecartus is the same as that in Yescarta, they have different manufacturing processes because while Yescarta does not involve the enrichment of T cells, Tecartus necessitates such an enrichment. Furthermore, february 2022 and april 2021 were the dates on which two BCMA-specific CAR-T cell therapies could successfully get approval for the treatment of a number of myelomas, i.e., ciltacabtagene autoleucel (Carvykti) and idecabtagene vicleucel (Abecma) (37). **Table 1** offers detailed information regarding all FDA-approved CAR-T cells therapies (51).

**Table 1:** Summary of FDA-approved CAR-Ts therapies.

Generic name	Tisagenlecleucel		Ciloleucel	Autoleucel	Maraleucel	Vicleucel	Autoleucel
Brand name	Kymriah		Axicabtagene	Brexucabtagene	Lisocabtagene	Idecabtagene	Ciltacabtagene
Company name	Novartis		Kite Yescarta	Kite Tecartus	Juno Breyanzi	Bluebird Abecma	J&J and Legend Carvykti
Approval’s date	1	2017	2017	2020	2021	2021	2022
	2	2018	2021	2021	2022	.	.
	3	2022	2022	.	.	.	.
Target antigen/ Antibody	CD19/ Mouse FMC63		CD19/ Mouse FMC63	CD19/ Mouse FMC63	CD19/ Mouse FMC63	BCMA/ Mouse BB2121	BCMA/ dual camel single-domain antibodies
Hinge/ transmembrane	CD8α/CD8α		CD8α/CD8α	CD28/CD28	IgG4/CD28	CD8α/CD8α	CD8α/CD8α
Costimulatory domains	4-1BB & CD3ζ		CD28 & CD3ζ	CD28 & CD3ζ	4-1BB& CD3ζ	4-1BB& CD3ζ	4-1BB & CD3ζ
Disease Approval	R/R CAYA B-ALL		R/R LBCL	R/R MCL	R/R LBCL	R/R MM	R/R MM
Vector/ promoter	Lentiviral/ EF1α		Gammaretroviral/ LTR	Gammaretroviral/ LTR	Lentiviral/ EF1α	Lentiviral/ MND	Lentiviral/ EF1α
Pivotal clinical trial	NCT02435849 ELIANA trial (39)		NCT02348216 ZUMA-1 (40)	NCT02601313 ZUMA-2 (41)	NCT02631044 Transcend NHL001 (42)	NCT03361748 KarMMa (43)	NCT03548207 CARTITUDE-1 (44)
	NCT02445248 JULIET trial (45)		NCT03105336 ZUMA-5 trial (46)	NCT02614066 ZUMA-3 trial (47)	NCT03575351 TRANSFORM trial (48)	.	.
	NCT03568461 ELARA trial (49)		NCT03391466 ZUMA-7 (50) trial	.	.	.	.
Outcomes	81% ORR		58% CR	67% CR	53% CR	33% CR	67% CR
	40%		74%	59%	.	.	.
	71%		65%	.	.	.	.
No. of Patients	75		111	74	269	128	97
	93		153	.	.	.	.
	98		180	.	.	.	.

CAYA R/R B-ALL, Children and young adults with Relapsed/Refractory Bcells-precursor Acute Lymphoblastic Leukemia; LBCL, Large B-Cell Lymphoma; MCL, Mantle Cells Lymphoma; MM, Multiple Myeloma; ORR, overall remission rate; CR, complete response.

## 5 Advances in CAR-T development: focus on off-the-shelf allogenic CAR-T

CAR-T therapy is an innovative treatment for certain cancers, especially hematologic malignancies, but traditional autologous CAR-T therapy has several limitations. Autologous CAR-T involves extracting and engineering T-cells from individual patients, making it time-consuming and expensive, with potential functional impairments in the engineered T-cells. Additionally, the process can take weeks, delaying treatment (52, 53). This main challenge has led to interest in off-the-shelf allogenic CAR-T cells (alloCAR-T), which use T-cells from a single healthy donor, offering faster availability, broader applicability, and cost-effectiveness. However, challenges remain, such as avoiding GVHD and host-mediated graft rejection (53, 54). Advances in genome-editing techniques and non-gene-editing technologies are helping to address these challenges, making alloCAR-T therapies a promising future treatment. Here, we have highlighted the progress in CAR-T development, particularly in engineering alloCAR-T cells.

### 5.1 Methods to engineer off-the-shelf allogenic CAR-T

As mentioned above, creating a universal off-the-shelf allo-CAR-T product requires overcoming key issues include preventing HvG, avoiding GvHD, preventing fratricide (where CAR-T cells attack each other), and enhancing the persistence of the infused allo-CAR-T cells. These challenges can be addressed through gene editing technologies and non-gene editing approaches. Additionally, supporting the expansion and persistence of CAR-T cells can be achieved by producing proinflammatory cytokines, such as IL-15 or IL-18, through transgenic methods (55, 56).

#### 5.1.1 Gene editing technology

Gene editing technologies have become crucial for modifying allogenic CAR-T cells to reduce immune rejection, enhance functionality, and improve safety. Methods like homing endonucleases (also called meganucleases) (57), transcription activator-like (TAL) effector nucleases (TALENs) (52, 58–60), zinc-finger nucleases (ZFNs) (61), and CRISPR-Cas9 (62), are used to create precise genetic changes. However, multiple modifications can increase the risk of off-target mutations and chromosomal translocations, with CRISPR-Cas9, TALENs, and ZFNs posing higher risks than newer techniques like base editing and prime editing (52, 63, 64). Additionally, making several edits to CAR-T cells could lead to other unwanted effects, such as reduced *in vivo* persistency (64). Homing endonucleases, while highly specific, are challenging to engineer, limiting their use (65). MegaTALs, which combine homing endonucleases with TAL effector arrays, offer enhanced specificity and editing efficiency (66). However, both homing endonucleases and MegaTALs require custom engineering for specific targeting, which limited their large-scale adoption of these gene-editing platforms (67). TALENs and ZFNs induce double-strand breaks (DSBs) to disrupt target genes, but are costly and time-consuming (68–73). Despite these challenges, TALEN-edited CAR-T cells have been used in clinical trials to knock out αβTCR and CD52 (52, 58, 59). CRISPR-Cas9, using guide RNAs, is more flexible, cost-effective, and precise, and is widely used in clinical trials for modifying CAR-T cells (68, 74–77). Newer techniques like base editing and prime editing, which do not induce DSBs, offer fewer off-target effects and are being tested in clinical trials for CAR-T therapies. These advancements hold promise for safer, more effective off-the-shelf allo-CAR-T treatments (64, 78, 79) (**Table 2**). Genome-edited allogeneic CAR-T cells offer therapeutic potential but pose risks like chromosomal abnormalities and secondary malignancies, requiring long-term monitoring. CRISPR/Cas9 and other editing techniques may cause unintended genetic changes, emphasizing the need for vigilant safety assessments (53).

**Table 2:** Gene-editing methods for manufacturing allogenic off-the-shelf CAR-Ts.

Gene-Editing Technology	Recognition Site	Modification pattern	Target sequence size	Advantage/ disadvantage	Delivery
ZFN	Zinc finger protein	Fok1 nuclease	9–18 bp	small size	Easy
TALEN	RVD tandem repeat region TALE protein	Fok1 nuclease	14–20 bp	large size	Difficult
CRISPR/Cas9	guideRNA and tracrRNA	Cas9 nuclease	20 bp- guide + PAM sequence	large size of SpCas9	Moderate to difficult
Base-Editing	Cas sequence + base-editor mRNA	Four possible transition mutations: C→T; A→G; T→C; G→A	CRISPR/Cas dependent	large site and added complexity	Difficult

#### 5.1.2 Non-gene editing approaches

Non-gene editing approaches to reduce GvHD in CAR-T therapy focus on inhibiting TCR signaling, using virus-specific T cells (VSTs), memory T cells, and γδ T cells. Truncated CD3 can inhibit TCR signaling to minimize GvHD while preserving CAR activity, though solid tumors present challenges due to antigen heterogeneity and immunosuppressive environments. VSTs, particularly those from HLA-matched donor libraries, have shown low GvHD risk, with CAR-transduced VSTs demonstrating anti-tumor effects. Another strategy is using emory T cells that being less alloreactive than naïve T cells, although their role in allogeneic CAR-T therapy remains underexplored. γδ T cells, which function independently of MHC complexes, provide a unique advantage by reducing GvHD and tumor escape risks. Overall, these non-gene editing strategies present safer, promising alternatives for improving CAR-T therapy efficacy while minimizing GvHD (80).

Although gene editing technologies have many advantages, their main challenge is safely removing multiple genes (multiplexing) while minimizing risks. As an alternative, non-gene-editing technologies provide an interesting approach to support the development of allogenic CAR-Ts in the future. Nowadays, three strategies have been developed as non-gene-edited approaches: 1) A TCR inhibitory molecule (TIM) that, when incorporated into T-cell DNA, competes with TCR elements, rendering the TCR unresponsive. This method was tested with an NKG2D-based CAR in metastatic colorectal cancer (81). 2) A miRNA scaffold targeting CD3ζ, which completely eliminates TCR expression, was assessed in a phase I clinical trial with a BCMA-targeting CAR-T for relapse/refractory multiple myeloma (82). 3) Third strategy involves retaining TCR/HLA-I in the endoplasmic reticulum (ER) to prevent GvHD and host-versus-graft (HvG) reactions. This is achieved by using a peptide like KDEL, combined with an scFv targeting the TCR, to retain all TCRs in the ER (83).

Removing the TCR from T-cells is beneficial, but factors influencing cellular persistence in an allogenic setting are still unclear. While HLA-I/II proteins play a role in HvG reactions, other factors like metabolic regulation may also affect persistence. Non-gene-editing approaches, such as combining miRNA or siRNA sequences to target multiple genes at once, make it easier to address these complexities (84–86). A new multiplex shRNA platform allows the targeting of up to four genes simultaneously, including those involved in GvHD (CD3ζ), HLA-I/HvG (B2M, CIITA), apoptosis (CD95), immune checkpoints (LAG-3), and co-stimulation, reduction/persistence (CD28) (87).

Gene-edited allogeneic CAR-T cells reduce GvHD risk through TCR knockout but face challenges like immune rejection, reduced persistence, and complex manufacturing. In contrast, non-edited CAR-T cells, including virus-specific, memory, and γδ T cells, offer safer alternatives with lower immune rejection and better persistence but may still pose some risk of allo-reactivity. While gene editing provides precise control, it also introduces regulatory and cost challenges. Non-edited approaches leverage natural T cell subsets for safety and efficacy, making them promising but requiring further optimization for tumor targeting (53).

### 5.2 Clinical experience with off-the-shelf allogenic CAR-T

#### 5.2.1 Achievements so far

The clinical development of off-the-shelf allogenic CAR-T therapies is still in its early stages, with no approvals yet. Early-phase trials show promising results, but challenges remain, including the risk of GvHD, graft rejection, and infections. Unlike autologous CAR-T treatments, which are costly and time-consuming due to patient-specific manufacturing, allogenic CAR-T therapies aim to offer a more accessible alternative, especially for patients who cannot afford personalized therapies. Progress has been slower than expected due to safety concerns and technical challenges in gene editing, though recent advancements are improving their potential. The first use of allogenic CAR T cells in humans was reported in 2017, with UCART19, a TALEN-edited, αβTCR/CD52-knockout product. It was evaluated in two Phase I studies (PALL and CALM) for relapsed or refractory B-cell acute lymphoblastic leukemia (R/R B-ALL) in pediatric and adult populations. The trials showed promising results, especially in infants, who were successfully bridged to hematopoietic stem cell transplantation (HSCT) (52, 58, 59). In 2020, combined data from two Phase I trials of UCART19 in children (7 patients) and adults (14 patients) with relapsed/refractory B-cell acute lymphoblastic leukemia (R/R B-ALL) demonstrated a promising complete response (CR/CRi) rate of 67%, although treatment-related deaths occurred (58). Updated results from the adult trial in 2022 (25 patients) showed an overall response rate (ORR) of 48%, with a median response duration of 7.4 months and median overall survival of 13.4 months. Importantly, GvHD was rare, with only two patients experiencing mild grade 1 acute skin-related GvHD (59) (**Supplementary Table 3**).

In the ALPHA and ALPHA2 studies, an anti-CD19 CAR-T therapy, a second dose of ALLO-501/ALLO-501A was given approximately 30 days after the first infusion to enhance the persistence of CAR-T cells in peripheral blood beyond day 28, aiming to improve response duration (88, 89). In 2023, a Phase I trial of ALLO-715, a TALEN-edited CAR-T product targeting BCMA for relapsed/refractory multiple myeloma (R/R MM), showed an overall response rate (ORR) of 55.8% among 43 patients. Although no cases of GvHD were reported, 88% of patients experienced grade ≥3 adverse events, and 53.5% had infections, with 23.3% experiencing severe infections, including 7% fatal cases (60). Additionally, ALLO-316, an anti-CD70 CAR-T product, is being tested in the Phase 1 TRAVERSE study for patients with advanced or metastatic clear cell renal cell carcinoma (CcRCC) (90). TALEN-edited CAR-T products like UCART123 (anti-CD123) and UCART22 (anti-CD22) are also being tested in Phase I trials for R/R B-ALL and AML (BALLI-01 and AMELI-01 studies respectively), with 70.8% of patients showing objective responses and no cases of GvHD (91, 92). A clinical hold was imposed on UCART123 in the ABC study following a fatality, resulting in dose adjustments and a recommendation to restrict the total cyclophosphamide dose (87, 93).

**Supplementary Table 3** summarizes key clinical trials applying different gene-editing and non-gene editing approaches in the development of allogeneic CAR-T cells therapies.

GRm13Z40-2 is a CAR-T cell therapy targeting the IL-13 receptor α2 (IL13Rα2) in glioblastoma patients. In its manufacturing, ZFNs were used to knock out the glucocorticoid receptor in T cells, enabling patients to receive high-dose glucocorticoids without compromising CAR T-cell function. The therapy was administered intracranial in four doses, along with recombinant IL-2 to enhance T-cell growth and persistence. The treatment was generally well tolerated, with no signs of GVHD. In terms of efficacy, 66% of patients showed temporary clinical responses, including tumor size reductions or necrosis (61).

The ARCUS genome-editing technology is used in the anti-CD19 allogenic CAR-T product PBCAR0191, currently being tested in a Phase I/II study for R/R non-Hodgkin’s lymphoma (R/R NHL) and B-ALL (NCT03666000). Another allogenic CAR-T product, PBCAR19B, designed to reduce immune rejection by host T-cells and NK cells, indicated a 71% ORR and a 43% CR rate in a Phase I study (NCT04649112) during 2021-2023 years in 13 patients (94).

CRISPR/Cas9 is being utilized in several promising CAR-T therapies for various cancers. CTX110, targeting CD19, is currently being evaluated in the Phase 1 CARBON trial for relapsed/refractory non-Hodgkin’s lymphoma (r/r NHL), demonstrating a 67% overall response rate (ORR) at the highest dose, with no GvHD despite significant HLA mismatch. A second infusion provided additional benefit (95, 96). CTX-130 (as an anti-CD70 CAR-T) has been tested in the COBALT-LYM (T-cell lymphoma) and COBALT-RCC (advanced clear cell renal cell carcinoma) studies. In T-cell lymphoma, the ORR was 71% with no GvHD, while in RCC, the ORR was 8%, though one patient achieved a durable complete response (97, 98). CB-010 (as an anti-CD19 CAR-T) with CRISPR-edited TRAC and PD-1 knockouts, demonstrated a 94% ORR in the ANTLER Phase 1 trial for B-NHL patients, which is comparable to autologous CAR-T therapies, and showed no GvHD (99). CRISPR-edited CD7-targeted CAR-T cells for T-ALL exhibited high efficacy, with complete response rates ranging from 71% to 91%, and no GvHD or neurotoxicity (62, 77). Early trials have also shown promising results for NK/T lymphomas and AML (75, 100).

However, alternative gene-editing methods have also been explored. P-BCMA-ALLO1, an allogenic CAR-T targeting BCMA for relapsed/refractory multiple myeloma (RRMM), is manufactured using the piggyBac^®^ DNA Delivery System and the Cas-CLOVER™ gene editing system, which eliminates TCR expression and reduces MHC class I expression to prevent GvHD. It showed strong activity in MM xenografts and is being tested in a Phase I study. P-MUC1C-ALLO1, targeting MUC1-C for common solid tumors, is also produced with piggyBac^®^ and Cas-CLOVER™, leading to TCR and MHC class I knockout for an enriched T stem cell memory product (101, 102). Another approach used base-pair editing in a Phase I trial of CD7 CAR-T cells. Preliminary results showed mixed outcomes, including one patient achieving leukemic remission, another undergoing stem cell transplantation, and a third developing a fatal fungal infection. Adverse events included cytokine release syndrome and multilineage cytopenia (79).

CAR-T products using non-gene-editing technologies have also been tested in clinical trials. CYAD-101, an allogenic CAR-T designed to co-express an NKG2D-based CAR and an inhibitory peptide to interfere with TCR signaling, was tested in the alloSHRINK Phase I study for unresectable metastatic colorectal cancer. After standard chemotherapy, 25 patients received three infusions of CYAD-101. There were no dose-limiting toxicities (DLT) or graft-versus-host disease (GvHD), and the overall response rate (ORR) was 13%, with 60% achieving stable disease (81, 103). CYAD-211, using a miRNA-based shRNA approach to silence the CD3ζ component of the TCR while co-expressing an anti-BCMA CAR, was tested in the IMMUNICY-1 Phase I trial for relapsed/refractory multiple myeloma. Early results from 12 patients showed 3 partial responses and 8 stable diseases, with no DLT, GvHD, or neurotoxicity (104). A CD19-targeting allogenic CAR-T, using intracellular retention to prevent TCR expression, was tested in a Phase I study for non-Hodgkin’s lymphoma, showing no GvHD and a 75% ORR in the first eight patients (83). Lastly, FT819, an iPSC-derived CD19-targeting CAR-T, was evaluated in a Phase I study for B-cell malignancies, showing a tolerable safety profile with no DLT or GvHD in 12 patients (105) (**Supplementary Table 3**).

## 6 The major challenges that must be resolved for the purpose of allogenic CAR-T therapy

In spite of their many favorable traits, a number of challenges are also ascribable to allogenic CAR-T cell. In fact, allogenic T cells may result in severe GVHD, which is predominantly caused by a TCR-mediated immune response to the life-threatening host tissues. In turn, the host immune system may also lead to the induction of allorejection, which can result in impeded anti-tumor activity and limited effectiveness of the therapy. This is attributable to the fact that through the detection of nonself- HLA class I and class II proteins found on the membrane of donor T-cells, the host immune cells can mainly detect and eliminate allogeneic cells. This is the primary objective of allogeneic CAR-Ts, which limits their duration of response and activity (38).

When designing allogeneic CAR-Ts, one of the most commonly used strategies to prevent GVHD is the creation of TCR-deficient T cells by employing genome editing tools, including as TALENs (106, 107), CRISPR/Cas9 (107, 108), and ZFN (3, 4).

A number of allogeneic candidates have attained the same objective response rates as the ones witnessed in their autologous counterparts on the clinical scale, and irrespective of two patients (one adult and one infant) presenting with acute skin GvHD Grade I, which was controlled easily (59), the preliminary data obtained from those investigations totally indicated no evidence of acute GvHD. As a result, in spite of earlier concerns, the modifications that were conducted in order to avoid GvHD in allogeneic ‘off-the-shelf’ CAR-Ts apparently suffice to decrease such a risk drastically. In addition, the strategies used to decrease allorejection are also analyzed, which test repeated rounds of infusion by employing chemotherapy-resistant CAR-Ts. This allows for deeper/prolonged lymphopenia or genetic elimination of the main molecules that govern the immunogenicity of the CAR-T cells (38).

For instance, the studies on CALM indicated that even though the expansion of CAR-T cells took place 8 to 14 days after injection, a rapid decline was experienced by most participants by day 28, which indicated a limited duration of response (109). Different lymphodepleting regimens were tested that showed the intensity was positively correlated with the cellular persistence; however, the risk of infections was increased, as observed in the UNIVERSAL investigation with a fifth-grade fungal pneumonia incident (60). In order to address this issue, the subsequent investigations suggested a second dose of 501A and ALLO-501 in order to maintain the levels of CAR-T (89). Nonetheless, the safety concerns resulted in dose adjustment recommendations in investigations such as ABC and incidents of grade 5 pneumonia associated with conditioning regimens (87).

One of the popular techniques employs genome editing of MHC class I proteins through the disruption of the β2-microglobulin (β2M) locus (38). The engineering of the PBCAR19B cells was targeted to knock-down(human β2M) and express a HLA-E transgene in order to avoid allorejection (87). The proof-of-concept provided by the preliminary record results indicated that such modifications were apparently successful in delaying the host NK-and T- cells’ recovery. In much the same way, it has been reported that CTX-130, which is a modified anti-CD70 allo -CAR-T used for the disruption of the CD70 and β2M genes so as to decrease the fratricide and allorejection, elicits a durable complete response in an individual suffering from Renal Cell Carcinoma (RCC) (110), This may indicate that, even in solid tumors, the technique is actually enhancing the allogeneic CAR-Ts’ activity. Currently, CB-011, which is an anti-BCMA allo-CAR-Ts engineered with CRISPR/CAS12a to knock out both TRAC and β2M while co-expressing a β2M-HLA-E fusion peptide, is under evaluation in the CaMMouflage Phase 1 trial and has presented encouraging preclinical data, resulting in noticeably enhanced durability of anti-tumor activity (87).

In addition, the creation of a bank of allogeneic T cells is also an alternative, which has been predominantly utilized for non-modified and virus-specific T cells but also for anti-CD123 retrovirally transduced CAR T for the purpose of treating acute myeloid leukemia (38, 111).

One of the big concerns is the safety risks associated with the application of gene-editing technologies. In one patient receiving a consolidation dose of ALLO-501A, a chromosomal abnormality was recorded, which resulted in stopping all the investigations with the same technology for several months (89). The studies found that the observed chromosomal abnormality was not associated with the TALEN gene manufacturing or editing process; however, it questioned the safety consideration of genome-edited cellular therapies.

In recent years, the FDA has noted a risk of T-cells malignancies, which is applicable to all currently authorized therapies using CAR-Ts. Such concerns are raised regarding autologous CAR T-cells that can persist for a long duration. Given that allogenic CAR-Ts lack persistence, such a safety concern does not apply at present. As the technology is enhanced progressively, the increased persistence of the CAR-T cells may result in the same safety concerns (37, 87).

## 7 Conclusions and future perspective

Comprehensive investigations are essential to establish the efficacy of allogeneic CAR-T therapies compared to approved autologous CAR-Ts, particularly for treating solid tumors with limited therapeutic options. Current findings suggest that allogeneic CAR-Ts have the potential to overcome significant barriers to access for a broader patient population. This advancement stems from innovative approaches that disrupt TCRs and mitigate GvHD, which poses a major toxicity risk in allogeneic T-cells therapies. Genetic TCRα ablation using targeted genome-editing techniques has gained popularity, yet challenges remain, including the risk of DNA double-strand breaks and complexities in manufacturing that may impact cell yield and fitness. Non-genome-editing tools present a promising alternative, offering potentially safer and more adaptable methods for developing next-generation CAR-Ts. Despite the encouraging prospects of these techniques in reducing GvHD risks, a critical challenge remains: managing the HvG reaction post-infusion. Therefore, it is imperative for “off-the-shelf” allogeneic CAR-Ts to address both GvHD and HvG reactions. This can be achieved through various modifications, such as the disruption or downregulation of genes involved in allorejection, including CIITA, CD52, and B2M.To enhance the development of allogeneic CAR-T cells, we recommend focusing on the following strategies:

- Investing in alternative genetic modifications that minimize GvHD and HvG risks.
- Exploring non-genome-editing approaches to simplify manufacturing and improve cell viability.
- Conducting preclinical and clinical trials to assess the safety and efficacy of modified allogeneic CAR-Ts in diverse patient populations.
- Developing robust monitoring systems to track post-infusion reactions and optimize patient management.

In conclusion, continued investment in innovative technologies is essential for the safe infusion of allogeneic CAR-T cells while enhancing their efficacy, persistence, and safety profiles.
