# Supplementary material for: Allogeneic CART progress: platforms, current progress and limitations
Source: Front Immunol. 2025 Jun 12;16:1557157. doi: 10.3389/fimmu.2025.1557157 (PMC12198129; doi:10.3389/fimmu.2025.1557157)
Supplement: Supplementary file 1 [file Table3.docx]

**Supplementary data file for**

**Allogeneic CART progress: platforms, current progress and limitations**

**Supplementary table 3.** **Overview of clinical trials utilizing engineered allogeneic CAR-T cell therapies categorized by gene-editing and non-gene editing** **technology).**

| **Gene**  **Engineering**  **Technology** | **Target Antigen** | **Disease** | **Generic Name** | **Lead Designers** | **Country** | **Trial Phase** | **Trial Number and ID** | **Year** | **Recruiting**  **Status** | **Outcome Measure** |
| --- | --- | --- | --- | --- | --- | --- | --- | --- | --- | --- |
| **TALEN**  **TALEN** | CD19 | CALL | ALLO 501/UCART19 | Cellectis S.A.; Allogene Therapeutics | France  USA | I | NCT02746952;  **CALM** | 2016 | Completed | Dose escalation phase: Occurrence of Dose Limiting Toxicities (DLTs).  Dose expansion phase: Adverse Events (AEs) observed throughout the study;  Incidence and severity of adverse events as indicators of safety and tolerability. |
|  |  | PALL | ALLO 501/UCART19 | Cellectis S.A.; Allogene Therapeutics | France  USA | I | NCT02808442;  **PALL** | 2016 | Completed | Frequency and intensity of adverse events; Rate of molecular remission. |
|  |  | R/R Large B Cell or Follicular Lymphoma | ALLO 501/UCART19 | Cellectis S.A.; Allogene Therapeutics | France  USA | I | NCT03939026;  **ALPHA** | 2019 | Primary Completion | Percentage of subjects developing Dose Limiting Toxicities at escalating doses of ALLO-501;  Percentage of patients experiencing Dose Limiting Toxicity with ALLO-647 when given in combination with fludarabine/cyclophosphamide prior to ALLO-501 administration. |
|  |  | R/R LBCL and CLL/SLL | ALLO-501A | Cellectis S.A.; Allogene Therapeutics | France  USA | I/II | NCT04416984;  **ALPHA 2** | 2020 | Recruiting | Dose-limiting toxicity is defined as adverse events related to ALLO-501A, as specified in the protocol, occurring within 28 days after infusion. |
|  | BCMA | R/R Multiple Myeloma | ALLO-715 | Allogene Therapeutics; Cellectis S.A. | USA  France | I | NCT04093596;  **UNIVERSAL** | 2019 | Recruiting | Dose-limiting toxicities are characterized as adverse events related to ALLO-715, occurring within 28 days after the infusion of ALLO-715. |
|  | CD70 | AML | ALLO-316 | Allogene Therapeutics | USA | I | NCT04696731;  **TRAVERSE** | 2021 | Recruiting | Percentage of subjects developing Dose Limiting Toxicities at escalating doses of ALLO-316;  Percentage of patients experiencing Dose Limiting Toxicity with ALLO-647 when given alongside fludarabine/cyclophosphamide before ALLO-316 administration. |
|  | CD123 | BPDCN | UCART123 | Cellectis S.A. | France | I | NCT03190278;  **AMELI-01** | 2017 | Completed | Safety of UCART123v1.2 – Frequency, type, and severity of adverse events (AEs) and serious adverse events (SAEs) during the study. |
|  |  |  |  |  |  |  | NCT04106076 | 2019 | Completed | Frequency, type, and severity of adverse events (AEs) and serious adverse events (SAEs) observed throughout the study. |
|  |  |  |  |  |  |  | NCT03203369;  **ABC123** | 2017 | Completed | Frequency, type, and intensity of adverse events (AEs) and serious adverse events (SAEs). |
|  | CD22 | B-ALL | UCART22 | Cellectis S.A. | France | I | NCT04150497;  **BALLI-01** | 2019 | Recruiting | Frequency, type, and severity of adverse events (AEs) and serious adverse events (SAEs) throughout the study in connection with UCART22 and/or lymphodepletion. |
|  | SLAMF7 | R/R Multiple Myeloma | UCARTCS1 | Cellectis S.A. | France | I | NCT04142619;  **MELANI-01** | 2019 | Completed | Frequency, type, and severity of adverse events (AEs) and serious adverse events (SAEs) throughout the course of the study. |
| **Zinc Finger**  **Nuclease (ZFN)** | IL13-zetakine | R/R Malignant Glioma | GRm13Z40-2 | City of Hope | USA | I/II | NCT01082926 | 2010 | Completed | Safety of GRm13Z40-2 CTL CNS loco-regional cellular immunotherapy |
| **ARCUS** | CD19 | R/​r NHL and R/​r B-cell ALL | PBCAR0191/Azercabtagene zapreleucel | Precision BioSciences | USA | I/II | NCT03666000 | 2019 | Recruiting | Phase 1 Dose Escalation/Phase 1b Dose Expansion: Frequency of Participants with Azer-cel related Adverse Events (AEs) defined as dose limiting toxicities (DLTs) |
|  |  | CD19-expressing Malignancies | PBCAR19B | Precision BioSciences |  | I | NCT04649112 | 2021 | Completed | To identify the maximum tolerated dose (MTD);  To evaluate adverse events as dose-limiting toxicities according to the protocol and CTCAE v5.0 criteria. |
|  | BCMA | R/R Multiple Myeloma | PBCAR269A | Precision BioSciences | USA | I | NCT04171843 | 2020 | Completed | Max Tolerated Dose (MTD) of PBCAR269A;  Number of Participants with Dose Limiting Toxicity(ies);  Objective Response Rate of Patients (ORR) |
|  | CD20 | r/​r NHL or r/​r CLL/​SLL | PBCAR20A | Precision BioSciences | USA | I/II | NCT04030195 | 2020 | Completed | Max Tolerated Dose (MTD);  Number of Participants With Dose-Limiting Toxicities;  Objective Response Rate (ORR); Progression-free Survival (PFS) |
| **CRISPR/Cas9** | CD19 | R/R B-Cell Malignancies | CTX110 | CRISPR Therapeutics | Switzerland | I/II | NCT04035434;  **CARBON** | 2019 | Recruiting | Duration of Response (DOR) for subjects with objective response events;  For B cell ALL, objective response rate (ORR) (complete remission + complete remission with incomplete blood count recovery) will be evaluated. |
|  |  | R/R NHL | CB-010 | Caribou Biosciences | USA | I | NCT04637763;  **ANTLER** | 2021 | Recruiting | Incidence of adverse events classified as dose-limiting toxicities occurring within 28 days following CB-010 infusion.  The primary endpoint is the objective response rate. |
|  |  | R/R B-ALL | CTA10 | Nanjing Bioheng Biotech | China | I | NCT04154709 | 2019 | Completed | Adverse events evaluated based on NCI-CTCAE v5.0 criteria;  Evaluation of MRD-negative overall response rate (MRD-ORR) at 3 months of treatment;  Evaluation of overall response rate (ORR = CR + CRi) at Months 6, 12, 18, and 24;  Assessment of event-free survival (EFS) at Months 6, 12, 18, and 24;  Evaluation of overall survival (OS) at Months 6, 12, 18, and 24. |
|  |  |  |  |  |  | I | NCT04227015 | 2020 | Primary Completion | Adverse events evaluated based on NCI-CTCAE v5.0 criteria;  Frequency of treatment-emergent adverse events [Safety and Tolerability]. |
|  |  | B-cell Malignancies | FT819 | Fate Therapeutics | USA | I | NCT04629729 | 2021 | Primary Completion | Incidence and characteristics of dose-limiting toxicities within each dose level cohort;  Incidence, type, and severity of adverse events (AEs) associated with FT819 as monotherapy and in combination with IL-2 in relapsed/refractory B-cell lymphoma, relapsed/refractory chronic lymphocytic leukemia, and relapsed/refractory precursor B-cell acute lymphoblastic leukemia. |
|  | CD19/CD7 | R/R B-cell Malignancies | GC502 | Gracell Biotechnologies | Chaina | I | NCT05105867 | 2021 | Completed | Incidence of dose-limiting toxicities as defined by the protocol;  Objective response rate following CAR-T infusion. |
|  | CD7 | R/R T-ALL/​LBL | WU CART 007 | Wugen | USA | I/II | NCT04984356 | 2022 | Completed | Incidence of adverse events associated with WU-CART-007 as assessed by CTCAE v5;  Maximum Tolerated Dose (MTD);  Composite Complete Response Rate;  Overall Survival;  Objective Response Rate;  Duration of Response;  Hematopoietic Stem Cell Transplant (HSCT) rate |
|  | CD70 | R/R Renal Cell Carcinoma | CTX130 | CRISPR Therapeutics | Switzerland | I | NCT04438083;  **COBALT-RCC** | 2020 | Recruiting | Part A (dose escalation): Incidence of adverse events;  Part B (cohort expansion): Objective response rate |
|  |  | R/R B Cell Malignancies | CTX130 | CRISPR Therapeutics | Switzerland | I | NCT04502446;  **COBALT-LYM** | 2020 | Recruiting | Part A (dose escalation): Incidence of adverse events  Part B (cohort expansion): Objective response rate |
| **Cas-CLOVER™** | BCMA | Multiple Myeloma (MM) | P-BCMA-ALLO1 | Poseida Therapeutics | USA | I | NCT04960579 | 2022 | Recruiting | Phase 1 Part 1: Evaluate the safety and maximum tolerated dose (MTD) of P-BCMA-ALLO1 based on dose-limiting toxicities (DLT).  Phase 1 Part 2: Assess the safety and tolerability of P-BCMA-ALLO1 when administered as a fixed cell dose.  Phase 1b: Investigate the impact of cell dose and study arm to inform the selection of the Recommended Phase 2 Dose (RP2D). |
|  | FKBP12; MUC1-C | Advanced or Metastatic Solid Tumors | P-MUC1C-ALLO1 | Poseida Therapeutics | USA | I | NCT05239143 | 2022 | Recruiting | Determine the maximum tolerated dose (MTD) and/or recommended phase 2 dose (RP2D) of P-MUC1C-ALLO1;  Assess the overall safety and tolerability profile of P-MUC1C-ALLO1;  Evaluate the preliminary efficacy of P-MUC1C-ALLO1. |
| **Base-pair editing** | CD7 | Relapsed T-Cell ALL | BE-CAR7 | Great Ormond Street Hospital | UK | I | ISRCTN15323014 | 2023 | Completed | Serious adverse events included cytokine release syndrome, multilineage cytopenia, and opportunistic infections.  The interim results of this Phase 1 study suggest that base-edited T cells warrant further investigation for patients with relapsed leukemia and highlight the expected risks associated with immunotherapy-related complications. |
| **Peptide-based**  **(TIM8)** | NKG2DL | Metastatic Colorectal Cancer | CYAD-101 | Celyad Oncology | Belgium | I | NCT03692429;  **AlloSHRINK** | 2018 | Primary Completion | Occurence of Dose Limiting Toxicities |
|  |  |  |  |  |  | I | NCT04991948;  **CYAD-101**-**002** | 2021 | Primary Completion | The Occurrence of Dose Limiting Toxicities (DLT) during the 'DLT reporting period';  The objective response rate (ORR) at the tumor assessment on Day 94. |
| **miRNA-based**  **shRNA** | BCMA | Multiple Myeloma | CYAD-211 | Celyad Oncology | Belgium | I | NCT04613557;  **IMMUNICY-1** | 2020 | Primary Completion | Occurrence of Dose Limiting Toxicities |
| **Non-gene editing** | CD19 | R/R B Cell Malignancies | ThisCART19 cells | Fundamenta Therapeutics | China | I | NCT04384393 | 2020 | Completed | Incidence of Dose Limiting Toxicities;  Complete Remission;  TRM: Treatment Related Mortality;  Objective Response Rate;  Duration of Response;  Incidence and Severity of Adverse Events as a Measure of Safety and Tolerability; Overall Survival Rate of 2 Years |
|  | CD19 | B-cell Malignancies | FT819 | Fate Therapeutics | USA | I | NCT04629729 | 2021 | Primary Compleon | Unknown. |
